# Reinvestigating the Neural Bases Involved in Speech Production of Stutterers: An ALE Meta-Analysis

**DOI:** 10.3390/brainsci12081030

**Published:** 2022-08-03

**Authors:** Ning Zhang, Yulong Yin, Yuchen Jiang, Chenxu Huang

**Affiliations:** 1 CAS Key Laboratory of Behavioral Science, Institute of Psychology, Chinese Academy of Sciences, Beijing 100101, China; 2 Department of Psychology, University of Chinese Academy of Sciences, Beijing 100049, China; 3 School of Psychology, Northwest Normal University, Lanzhou 730070, China; yinhaomin2486144@nwnu.edu.cn; 4 Department of Psychology, Renmin University of China, Beijing 100086, China; 2018102356@ruc.edu.cn; 5 School of Arts, Renmin University of China, Beijing 100086, China; 2019201523@ruc.edu.cn

**Keywords:** stuttering, neural bases, meta-analysis, activation likelihood estimation

## Abstract

Background: Stuttering is characterized by dysfluency and difficulty in speech production. Previous research has found abnormalities in the neural function of various brain areas during speech production tasks. However, the cognitive neural mechanism of stuttering has still not been fully determined. Method: Activation likelihood estimation analysis was performed to provide neural imaging evidence on neural bases by reanalyzing published studies. Results: Our analysis revealed overactivation in the bilateral posterior superior temporal gyrus, inferior frontal gyrus, medial frontal gyrus, precentral gyrus, postcentral gyrus, basal ganglia, and cerebellum, and deactivation in the anterior superior temporal gyrus and middle temporal gyrus among the stutterers. The overactivated regions might indicate a greater demand in feedforward planning in speech production, while the deactivated regions might indicate dysfunction in the auditory feedback system among stutterers. Conclusions: Our findings provide updated and direct evidence on the multi-level impairment (feedforward and feedback systems) of stutterers during speech production and show that the corresponding neural bases were differentiated.

## 1. Introduction

Human communication is facilitated by fluent speech activity. However, approximately 1% of the population worldwide have dysfluent speech production, such as repetitions, hesitations, prolongations or blocks [1,2]. Stuttering is a developmental disorder that occurs at between the ages of 2.5 and 6 years old. In comparison with people who do not stutter (PWNS), people who stutter (PWS) usually have abnormal prosody and disfluency when producing long words or complex sentences [3]. Stuttering affects an individual’s development of language and cognition, as well as self-cognition, emotion and social experiences [4,5,6]. Much interest has been received from various disciplines.

Researchers proposed the three-factor causal model [7] (also known as the P&A model) to account for the causes of stuttering from three perspectives: (1) impaired neural function; (2) triggers (e.g., variable syllabic stress and linguistic complexity); and (3) modulating factors (e.g., physiological arousal or individual readiness). Among the three factors, impaired neural function plays a vital role in stuttering. The multifactorial dynamic pathways (MDP) model was recently proposed [8], which considers that genetic/epigenetic, motoric, linguistic, emotional, and neural factors interact and, thus, lead to the onset or persistence of stuttering from preschool years onwards.

For stuttering intervention in early childhood, researchers have proposed the demands and capacities model (DCM), which takes into account that many factors are involved in the onset and development of stuttering, and none of them are pathological [9,10]. This model aims to train parents on how to decrease the relevant motoric, linguistic, emotional or cognitive demands on the child that might trigger their stuttering, thus resulting in the multi-domain capacities of the child being built to improve speech fluency [11,12]. 

In the past few decades, numerous studies have provided evidence on impaired neural function in speech production among PWS [13,14,15,16,17,18,19,20,21,22]. An ALE meta-analysis on the distinct brain activation patterns of persistent developmental stuttering revealed a right lateralization, with overactivation in the motor areas and cerebellum and decreased activation in the auditory areas [23]. Researchers also found that, in comparison with fluent speakers, PWS among adults had an abnormal volume of planum temporale and that its volume was positively related with the severity of stuttering [24,25,26,27]. Moreover, the supplementary motor area (SMA) has also been associated with stuttering, due to its function in the preparation and control of complex sequence motor planning [28]. Before articulation, the planned lexico-semantic and phonological representations are encoded into phonetic forms and motor sequences [29]. As a consequence, PWS might not execute syllabication and articulation successfully due to dysfunction in the SMA [28]. This view is consistent with the covert repair hypothesis [30], which considers that phonetic and motor planning failure would induce self-repairing, but unsuccessful repairing would lead to dysfluency [31,32,33,34].

A neurocomputational model—directions into velocities of articulators (DIVA)—specified the relationship between the behavioral and neural underpinnings of stuttering [35,36]. This model proposes that speech production contains feedforward and sensory feedback control processes. The former system is formed by the articulation circuit and initiation circuit. The two circuits are responsible for generating timed and coordinated muscle motor programs and turning this motor program on and off at the appropriate instances in time, respectively [37]. In the framework of the DIVA model, the initiation circuit involves the cortico-basal ganglia-thalamocortical loop, and the primary impairment among stutterers is dysfunction in the cortico-basal ganglia-thalamocortical loop. For the feedback process, the corticostriatum projection between the striatum and the auditory and motor cortices plays a critical role. The striatum detects a mismatch when the auditory feedback violates the expectation of the produced speech, thus leading to more competition between the motor programs and dysfluency [35,36,37]. Similarly, the state feedback control (SFC) theory proposes that the premotor regions are associated with generating forward models and receiving sensory feedback across the time course of speech production [38,39]. Online feedback control is internally learned and maintained based on previously processed associations between motor planning and sensory outcomes, and this feedback system can, in turn, correct the prediction mismatch errors [40]. Previous neuroimaging research has provided solid evidence for these two theories. In a picture naming study [19], deactivation in the superior temporal gyrus (STG) was found among stutterers; thus, the connections between the bilateral posterior superior temporal gyrus (pSTG) and putamen and thalamus among stutterers were weaker than those among non-stutterers.

To our knowledge, the most recent ALE meta-analysis on the neural bases of stuttering was published in 2017, in which, the trait (being a PWS) from the state of stuttering (the act of stuttering) was differentiated. Trait stuttering was characterized by right lateralization in the language processing areas and state stuttering was associated with overactivation in the right larynx and lip motor cortex [41,42]. However, PWS not only have disorders in speech production, but also in speech perception [15,43,44,45,46], which might, in turn, reflect the dysfunction of the auditory feedback system. Previous studies have shown that PWS exhibited more activation in the left anterior cingulate gyrus, left inferior frontal gyrus (IFG), and bilateral cerebellum in speech perception or comprehension tasks [18,20,44,46], even after therapy [47,48]. However, a previous meta-analysis did not differentiate whether the feedforward or feedback system was impaired in PWS and the neural bases for the two systems. Thus, it is necessary to perform an updated analysis to investigate this issue.

In the current meta-analysis, we tried to determine the fundamental brain activity patterns among PWS and PWNS by including up-to-date neuroimaging studies. We hypothesized that the meta-analysis would reveal evidence in the neurocomputational models, for instance, the decreased activity in the auditory regions and increased activity in the striatum.

## 2. Methods

### 2.1. Literature Search

Papers for analyzing contrasts between stutterers and non-stutterers in language processing were searched in Web of Science (www.webofscience.com) and PubMed (https://pubmed.ncbi.nlm.nih.gov/) databases (accessed on 15 February 2022). The search included papers published between January 1980 and February 2022. Search terms were “word production” OR “speech production” OR “language production” AND “stutter” OR “stutterer” AND “fMRI” OR “functional magnetic resonance imaging” OR “neuroimaging” OR “functional MRI” OR “functional imaging” OR “functional magnetic imaging”. This yielded 137 articles, which were then carefully reviewed and coded by two authors (N.Z and Y.Y) and were included in the meta-analysis if they met the following criteria: (1) the utilization of a task-related fMRI or PET method, not a fNIRS, ERP or MEG method, etc.; (2) reporting peaks of significant activation in standard stereotactic coordinates (Talairach or MNI space; those reporting only functional connectivity or correlation results were not included); (3) articles written in English; (4) performance of a language production task in the participants’ native language (not in a second language); (5) each study recruited two groups of healthy adult participants, (i.e., not children or adults with neurological or psychiatric disorders); and (6) not case studies (these were excluded). The two independent coders separately reviewed and coded the studies, and then, their coding was compared. Different coding for the same article was reviewed again to ensure no varied coding was generated. A final set of 24 articles was included in the meta-analysis.

**Figure 1:**
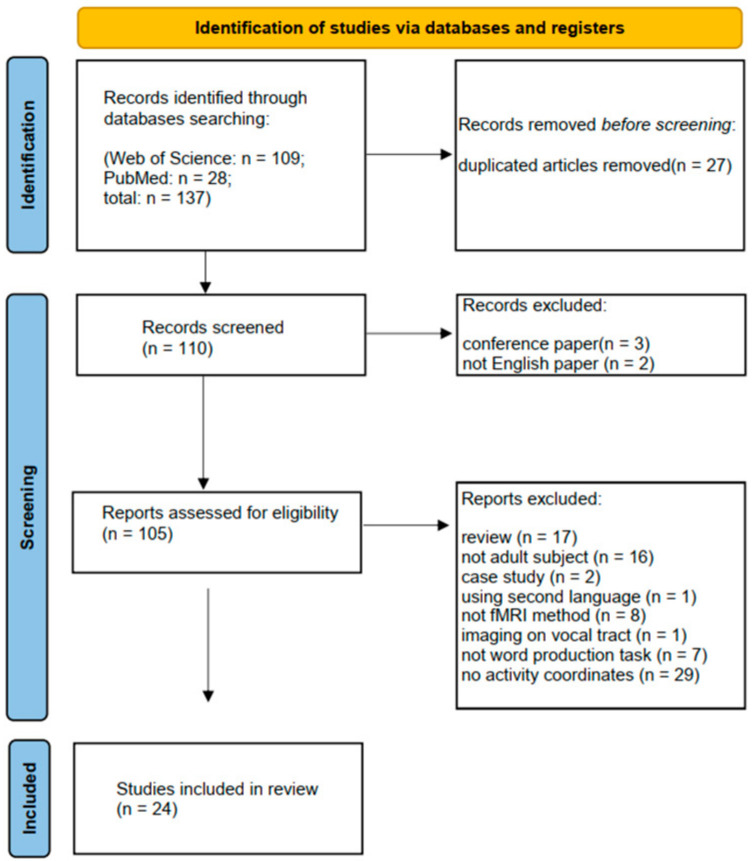
Flow diagram for identification and selection of studies.

The protocol for this systematic review was registered on the Prospective Register for Systematic Reviews (PROSPERO; registration ID: CRD42022336594). Identification, screening, eligibility, and inclusion procedures followed the Preferred Reporting Items for Systematic Reviews and Meta-Analyses (PRISMA) 2020 flow diagram. Figure 1 shows the flow diagram for the identification and selection of studies.

A minimum of 17 experiments were needed to be surpassed or achieved by the volume of a data set for each ALE meta-analysis, and a quantity lower than that would fail to promise a stable performance of the ALE algorism or reliable results [49]. Since one study may report experimental contrasts suitable for different conditions or may only report a contrast between two groups, contrasts were retrieved and re-categorized to achieve enough experiments for the following analysis. Some studies reported the results of the two groups independently, while some studies only reported a contrast between PWNS > PWS and PWS > PWNS. We combined the results of the PWNS group and PWNS > PWS as set A, and the PWS group and PWS > PWNS as set B. The two sets contained 21 and 26 experiments, and 326 and 355 participants, respectively (see Table 1 for details).

### 2.2. Activation Likelihood Estimation

ALE analyses were performed on the two foci sets of the non-stutter and stutter groups independently using GingerALE (version 3.0.2, The Brainmap Project, San Antonio, TX, USA, www.brainmap.org, accessed on 15 February 2022) [63,64,65]. The Talairach coordinates were transformed into MNI space using the icbm2tal tool provided by GingerALE [66]. Each activation peak is modelled as a probability distribution centered on the peak coordinates, generated by Gaussian smoothing. Statistical analysis of the transformed foci was validated using the Monte Carlo simulation (1000 permutations) with a cluster-forming voxel-level threshold at uncorrected *p* < 0.001 combined with cluster size correction using family-wise error (FWE) at *p* < 0.05 [49]. 

Conjunction and contrast analyses were performed using the contrast studies function in GingerALE to determine whether the non-stutter and stutter groups yielded discrepant patterns of neural underpinnings in language cognition. The two ALE maps of the two groups were pooled and subtraction analyses were conducted. The threshold of the results of the subtraction analyses was set to *p* < 0.001, with *p*-value permutations set to 10,000, and the minimum volume of clusters set to 200 mm^3^. 

The resulting ALE maps were displayed using multi-slice views generated using Mango software (developed by Jack L. Lancaster, Ph.D. and Michael J. Martinez, San Antonio, TX, USA, http://ric.uthscsa.edu/mango/, accessed on 15 February 2022) and the high-resolution MNI-space Colin 27 template provided by GingerALE (version 3.0.2, The Brainmap Project, San Antonio, TX, USA, www.brainmap.org, accessed on 15 February 2022).

The current study was approved by the Institutional Review Board of the Institute of Psychology, Chinese Academy of Sciences, and was conducted in accordance with the ethical principles of the Declaration of Helsinki.

## 3. Results

### 3.1. PWNS Group

**Figure 2:**
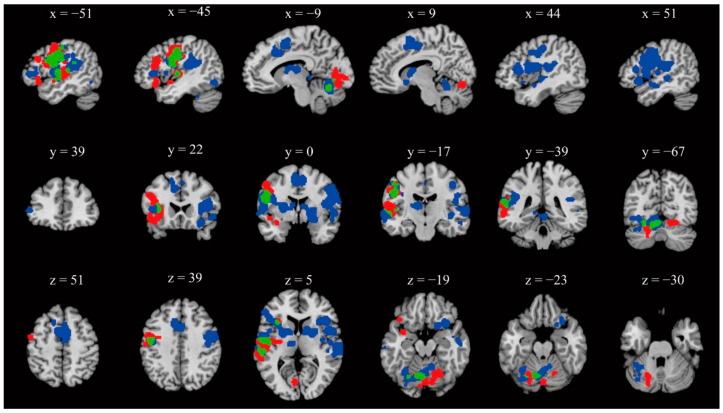
Activation likelihood maps for analyses of PWNS group (red), PWS group (blue) and their overlap (green) (uncorrected *p* < 0.001).

The ALE analysis of the PWNS group revealed significant clusters in the left hemisphere of the brain, with the Broca′s and Wernicke′s areas being located the most notable cluster (Figure 2, Table 2). Other regions included the remaining parts of the frontal lobe: left precentral gyrus and middle frontal gyrus (MFG); temporal lobe: left STG and middle temporal gyrus (MTG); parietal lobe: left postcentral gyrus; occipital lobe: left lingual gyrus; sub-lobar areas: left insula, claustrum and putamen; and part of the cerebellum: bilateral declive, left fastigium, culmen, uvula and tonsil.

### 3.2. PWS Group

For the PWS group, the ALE analysis revealed more regions than the PWNS group (Figure 2, Table 3). The most notable clusters included brain areas in the right hemisphere: the right postcentral and precentral gyrus, IFG, STG, MTG, transverse temporal gyrus, insula, putamen, claustrum, caudate, and inferior parietal lobule (IPL). Other clusters included the remaining parts of the frontal lobe: left IFG, left superior frontal gyrus, bilateral medial frontal gyrus, left precentral gyrus, and right paracentral lobule; temporal lobe: left STG, MTG, and left fusiform gyrus; parietal lobe: left postcentral gyrus and left IPL; sub-lobar areas: left insula, putamen, thalamus, cingulate gyrus, and parahippocampal gyrus; cerebellum: bilateral culmen, left declive, uvula, culmen, and cerebellar lingual.

### 3.3. Overlap between PWNS and PWS Group

Maps for the overlap between the two groups were generated after we delineated the maps for each group independently. The results showed that significant overlapping regions included the left precentral gyrus, IFG, pSTG, insula, and declive and fastigium in the cerebellum (Figure 2, Table 4). Moreover, conjunction analysis also revealed that the PWS group activated more brain areas than the PWNS group at the bilateral putamen, bilateral insula, bilateral precentral gyrus, right IFG, right STG, left postcentral gyrus, right caudate, right claustrum, right cingulate gyrus, and right claustrum. However, the results also revealed more deactivation in the left pSTG and MTG among the PWS group.

## 4. Discussion

In the current study, we re-investigated the neural bases of stuttering using an ALE meta-analysis. In comparison with previous meta-analyses [23,42], up-to-date results were presented. For non-stutterers, a general speech production network was found, including the left IFG, STG, MTG, precentral gyrus, postcentral gyrus, lingual gyrus, and insula. In comparison, overactivation in the right hemisphere including the IFG, pSTG, bilateral striatum, insula, precentral and postcentral gyrus, and cerebellum, and deactivation in the left aSTG and MTG were found among the PWS group.

The neural mechanism of speech production among PWS was different from that of non-stutterers. In the following, we discuss our results from the perspectives of the general network of speech production, the uniqueness of the neural mechanism of speech production among PWS and the role of the cerebellum in speech production.

### 4.1. General Network of Speech Production

The most notable findings were that the left IFG, STG and MTG were significantly activated brain areas among the two groups. These three areas, according to numerous studies, play vital role in speech production. Spoken word production involves a series of planning stages, i.e., conceptual preparation, lexical selection, phonological encoding, phonetic encoding, and articulation [29]. The left STG may be associated with semantic retrieval, but the anatomical regions in the STG might differ from the posterior STG, which is responsible for the retrieval of sematic representations, as well as the anterior STG, which is associated with primary auditory processing [67,68]. The left IFG may be associated with both sematic processing and post-phonological processing, e.g., syllabication and motor planning for articulation [67,68]. More importantly, these areas are overlapped with the domain general cognition network, suggesting that word production is a process which involves a large network of different areas.

In the language production model [29,67], semantic retrieval and phonological encoding are the two core processes in the planning stage before articulation. Previous fMRI studies on word production used different tasks or paradigms, in which different processing phases were involved. For instance, picture naming involves visual recognition before semantic retrieval, and the reading aloud task involves visual word recognition and grapheme to phoneme encoding. However, the shared processing of word production by differing tasks remained the same. Previous researchers performed a series of meta-analyses and systematic reviews on the neural bases of word production [67,68,69]. We discuss the brain areas responsible for different processes separately.

For semantic retrieval, previous studies found consistent results for activation of the left pSTG associated with semantic retrieval. de Zubicaray et al. [70] investigated word production using the picture–word–interference paradigm, in which a related word was presented on the picture (for instance, the word “apple” was presented on a picture of a banana), and the speaker was required to name the picture and ignore the distractor word. The results found overactivation in the left pSTG under semantically related conditions compared to neutral (non-related) conditions. Similar findings provided solid and consistent evidence for the role of the left pSTG in semantic retrieval [71,72,73]. Our results showed activation in the left pSTG in both groups, which provided direct evidence for its vital role in word production. Moreover, activation of the frontal cortices was also reported to be associated with semantic processing. The left IFG was reported to have stronger activation under semantic interference conditions [70,74,75,76]. Specifically, Abel and colleagues found that the activation level of IFG was positively correlated with the level of semantic interference [74,77]. The results of the current study are consistent with previous findings in that the left IFG and the pSTG were contained in the most notable cluster, which provided solid support for the crucial role of these regions in word production.

For phonological encoding, de Zubicaray and colleagues [78,79] investigated the phonological facilitation effect in word production (i.e., faster reaction time when naming a picture with a phonologically related distractor than an unrelated one), and the results showed stronger activation in the left aSTG. Similarly, Abel et al. [74,77] found stronger activation in the bilateral aSTG for the phonological facilitation effect. These findings suggest that the aSTG is a crucial region associated with phonological encoding. Moreover, activation in the left insula, bilateral supramarginal gyrus, and inferior parietal lobe was also found to be associated with phonological retrieval [80,81,82]. After the retrieval of phonological representation, motor planning and articulation proceed. The precentral gyrus was associated with articulation planning [67,68]. Rizio et al. [83] considered that the retrieval of the motor sequence of phonological representations, i.e., phonetic encoding, activates the precentral gyrus and its activation level is associated with phonetic encoding difficulty. The aSTG and precentral gyrus were involved in the most notable cluster in the current meta-analysis, which provided evidence on the significant role of the two regions in phonological encoding and motor planning [67,68,69,84].

In addition, domain general processes also play vital roles before the target lemma is selected and articulated. For instance, to solve the semantic interference, the non-targets had to be inhibited to ensure the target could be selected successfully. Therefore, the anterior cingulate cortices, orbitomedial prefrontal cortex, and inferior parietal lobe are activated due to their functions in conflict detection, task demand detection and updating, and inhibition of conflict response, respectively [70]. In the study by Criaud and Boulinguez [85], the go/no-go task elicited stronger activation of the insula, dorsal PFC, IFG, IPL and basal ganglia, which were the core nodes of the frontal-striatal network and were responsible for response inhibition. In addition, the basal ganglia are functionally connected to the medFG as part of the neural circuit associated with preparation and execution of movement [86]. These regions were found to be significantly activated in the current meta-analysis, which provided evidence that domain general cognitions are involved in word production.

### 4.2. Distinct Neural Underpinnings in PWS

Our results in the PWS group revealed greater activation in both the cortical and sub-cortical regions in word production than PWNS, among which, the STG and MTG, IFG and medial frontal gyrus, precentral and postcentral gyrus, insula, and basal ganglia were significantly involved.

The cognitive functions of MTG, pSTG and aSTG, as mentioned previously, were associated with phonological encoding and semantic retrieval in speech production and primary auditory processing, respectively. For activation in the aSTG, previous research has shown that bilateral aSTG is involved in auditory attention and conscious awareness of auditory stimuli [87,88,89,90]. In speech production, part of the self-monitoring is to receive the auditory input of produced speech. In comparison with PWNS, the dysfluent speech of PWS would cause more activation of the bilateral aSTG due to the cognitive load. For MTG, the left and right homologue might have varied cognitive functions. In comparison with primary auditory processing in the aSTG, researchers considered MTG to be related with higher level processing of auditory stimuli [91,92,93]. Previous research found greater activation in the bilateral MTG in both speech comprehension and production tasks [18,62], which suggested that the recruitment of the left MTG might reflect a higher level auditory processing of feedback, especially in terms of sensory integration [62]. In the current study, significant activation in the left MTG was detected in the PWNS, but no activation in this region was detected in the PWS. This finding suggests distinct mechanisms in auditory feedback processing among the two groups, with PWS being less proficient, which might be the crucial reason for stuttering.

A previous meta-analysis found deactivation of the STG among the PWS group [41], which is also a neural signature of stuttering. The anatomical pathway between auditory areas and the IFG was found to be reduced bilaterally in PWS, which suggested a dysfunction in feedforward processing during speech production [51,60,94]. However, since the STG could be subdivided into anterior and posterior regions, and the two regions were associated with different cognitive functions, it is necessary to specify the relationship between the deactivation in the different parts of the STG and stuttering. In the current study, as Figure 2 shows, overactivation in the pSTG and deactivation in the aSTG were found in the PWS group, which provided solid support for the distinct functions in the two parts of the STG. The cognitive demand in speech production among the PWS group might be higher than among non-stutterers, resulting in the overactivation of these regions as compensations. It can be inferred that the stutterers could retrieve the semantic and phonological representations with greater difficulty but still successfully, which indicated successful feedforward processing. In comparison, the deactivation in the aSTG might suggest a failure in feedback processing. Together with the finding of deactivation in the MTG among the PWS group, our results provide evidence for the dysfunction in auditory feedback processing in PWS.

The feedback processing recruits not only the aSTG and MTG—previous studies have found that PWS activates more brain areas in the right hemisphere as compensation [23,42]. According to the models of stuttering proposed by Giraud [48], when PWS could not speak fluently and made repetitions or blocks, the auditory system would send a feedback to suppress this activity, i.e., feedforward suppression from the motor cortex (precentral gyrus) to the aSTG. Moreover, for the speaker, a mismatch of prediction induced by the actual auditory inputs that were not the planned speech elicits greater activation of the IFG, since part of its cognitive functions is feedback monitoring and the release of speech plans [95]. Meanwhile, due to the impaired connection between the left IFG and precentral gyrus, the right homologue of the IFG and precentral gyrus would be activated as compensation [48]. According to previous reviews [23,42], activation of the right IFG and insula has been considered as “neural signature” of stuttering. One of the explanations of the overactivity of the right hemisphere is that it is associated with an inhibitory or stopping process [57]. Since the left IFG might not fulfill the feedback of suppressing, the right homologue shoulders the demand. Further investigation is still needed to provide more evidence.

Another important finding was that PWS recruited more basal ganglia in speech production than PWNS. Dysfunction in the basal ganglia might affect timing in speech production [96]. Execution of the motor sequences of speech requires exact timing of each muscular movement. Previous studies found that PWS would speak more fluently when speaking to the pace of a metronome, i.e., the rhythm effect [22,53]. The study of Lehéricy [97] found that the activity of the caudate was decreased in the planning stage of speech production but was increased when required to maintain the speed in execution. Similarly, Giraud [48] found a positive correlation with stuttering severity in the activity of the caudate and a trend of negative correlation in the speech naturalness. In addition, as mentioned in the previous section, the connectivity between the basal ganglia and medFG was associated with the preparation and execution of movements. As a consequence of dysfunction in motor execution, greater activity in the basal ganglia and medFG was shown, which was consistent with previous findings. Studies in music perception also found activation of the basal ganglia in rhythm or beat perception [98,99,100]. These findings provide evidence that the basal ganglia play a vital role in the domain general functions, and that language and music processing shared similar mechanisms, especially in terms of timing cognition.

It is worth noting that the activation of the postcentral gyrus in the PWS group was stronger than that in the control group. Previous studies have found close associations between basal ganglia (mostly caudate) and postcentral gyrus in both healthy subjects [48] and PWS [101,102]. A meta-analytic functional connectivity study [103] showed that the caudate has a strong connection with the bilateral precentral gyrus and postcentral gyrus in action and perception behavior. Still, the exact function of the connection between the basal ganglia, and the precentral and postcentral gyrus need further research and elucidation.

In summary, our results have updated the findings of previous meta-analyses in that the two regions in the STG were distinct in terms of activity patterns in the PWS group, and that the deactivation of the aSTG and MTG serves as crucial evidence of the dysfunction in auditory feedback processing among the PWS group.

### 4.3. The Role of Cerebellum in Speech Processing

The cognitive functions of the cerebellum in language processing have attracted much attention over the past few decades. Systematic reviews have been updating related findings [104,105,106,107]. Studies on language processing have found activity in the cerebellum across various linguistic components: phonological [108,109,110], lexico-sematic [111,112], semantic [113,114], syntactic [115,116], and discourse [117]. In a study [111] where participants were required to finish a word-stem completion task, greater activity in the right cerebellum was found when the word-stem had fewer completions (higher task difficulty condition). Moreover, besides its association with language processing, research has found that cerebellar activity may reflect the involvement of working memory, execution functions, visuospatial processing, emotional regulation, etc. [118]. This suggests that the role of the cerebellum might not be associated with domain-specific processing, but more with domain general functions.

The cerebellum is considered a fundamental site for motor control [119,120,121,122]. Researchers have assumed that the circuit between the cerebellum and motor cortex plays a vital role in motor learning and execution [121,123,124]. Courchesne and Allen [125] proposed that “the cerebellum predicts and prepares the internal conditions required for sensory, motor, autonomic, memory-related, attention-related, or linguistic operations, by acquiring the predictive relationships among temporally ordered multidimensional sequences of exogenously and endogenously derived neural activities”. A rest state functional connectivity study found that PWS showed abnormal connectivity between the cerebellum and the motor and prefrontal areas [126], which might lead to difficulty in lexical retrieval and motor executions of articulation.

It can be seen that the cerebellar functions are far more complicated and might work within varied networks with other brain areas. Our results were consistent with previous findings in that PWS showed greater activity in the cerebellum during language processing. However, further connectivity research is needed to specify the exact functions of the networks of the cerebellum and other areas.

### 4.4. Limitations and Future Directions

The current research focused on the neural bases of stuttering by performing an ALE meta-analysis of previously published neuroimaging studies, and the most notable results provided evidence for the dysfunction of auditory feedback processing among the PWS group. Since previous neuroimaging studies have recruited various types of participants, the individual differences (for instance, severity of stuttering, age, whether they received therapy and languages, etc.) might affect the results. However, a meta-analysis could not differentiate these effects. In addition, the studies included in the analysis varied in their quality. The number of foci varied across the studies, which had an effect on finding the clusters of the meta-analysis. By checking the results, it was found that different studies contributed a varied number of foci to finding the clusters, and there was even one study with no contribution of any foci. Future research should formulate uniform inclusion and exclusion criteria for ALE meta-analysis studies.

Moreover, another limitation of the current meta-analysis is the risk of bias. Firstly, we were unable to access data from unpublished research or studies that did not report all of the activated brain regions, especially those excluded studies reporting functional connectivity and correlation results. Secondly, studies that were not published in English were not able to be included. Thirdly, there is a trend that researchers choose not to publish studies without significant results, and this “bias against null results” plays a vital role in revealing the neural mechanism of human behavior [127]. While it is not easy for these problems to be solved currently, pre-print reports and promotion of open science may help with this issue.

Still, our findings presented a relatively clear picture of the neural underpinnings of stuttering, which provided applicative value in the intervention of stuttering. With the help of mature and precision medical technology, for instance, transcranial magnetic stimulation (TMS) and deep brain stimulation (DBS), it might be possible to intervene and modify the brain activity patterns of stutterers to improve their speech fluency.

## 5. Conclusions

The current study provided updated information on the shared and distinct neural bases of speech production between stutterers and non-stutterers using an ALE meta-analysis. Consistent with previous findings, this study revealed overactivity in the bilateral IFG, pSTG, basal ganglia, insula, precentral and postcentral gyrus, and cerebellum, and deactivation in the left aSTG and MTG among the PWS group. Based on the directions into velocities of articulators model and state feedback control theory, our findings provided support for the greater demand in the feedforward system in speech production. More importantly, solid evidence was provided for the dysfunction in the auditory feedback system, which might be the crucial cause of stuttering. These findings suggest that abnormal neural underpinnings of PWS were a result of dysfunction in various cortical–subcortical–cerebellar networks.

## Figures and Tables

**Table 1:** Details of experiments included in the ALE analysis of PWS and PWNS group.

First Author of Publication	Comparison	# Subjects		# Foci	
		PWS	PWNS	PWS	PWNS
Braun [13]	Orolaryngeal motor task > Rest Dysfluent language > Motor Fluent language > Motor	18	20	46	22
de Nil [43]	Oral reading > silent reading	10	10	4	4
Fox [14]	Speech-motor positive correlations with syllable rate	10	10	31	12
de Nil [44]	Oral reading > silent reading Pre-, post- and 1-year after treatment (3 exp)	13	10	33	8
Neumann [15]	Overt reading After therapy > before	5	16	35	9
Preibisch [16]	Overt reading > viewing meaningless signs	16	16	13	3
de Nil [18]	Repeating words > passive listening	15	15	19	15
Watkins [50]	Production with fluency or auditory feedback	10	10	11	21
Chang [51]	Speech production > non-speech production	20	20	27	59
Kell [47]	Overt > covert reading	26	13	32	0
Lu [19]	Covert picture naming > passive viewing	9	9	46	46
Sakai [52]	Speech production with auditory feedback > passive reading Speech production with auditory feedback > delayed feedback	8	10	14	18
Toyomura [53]	Reading sentences with auditory stimulus (rhythm/chorus) > solo	12	12	33	8
Howell [54]	Producing rising or falling tones	9	9	14	2
Ingham [55]	Overtly reading texts > monologue Monologue > overtly reading texts	18	12	17	20
Toyomura [56]	Oral reading > listening Oral reading > rest	10	10	22	9
Ward [20]	Picture describing > rest Sentence reading > rest	17	17	38	6
Lu [46]	Picture naming > rest	13	13	1	0
Neef [57]	Covert speaking > rest Covert humming a melody > rest	15	17	3	6
Lu [58]	Post-intervention > pre-intervention	26	13	3	0
Yang [59]	Stutter state anxiety > rest	19	19	19	2
Connally [60]	Speech production > rest	17	17	30	16
Neumann [61]	Linguistic prosody > neutral prosody Emotional prosody > neutral prosody	26	13	36	0
Sares [62]	Shifted pitch > unshifted pitch	13	15	0	3

**Table 2:** PWNS clusters found by ALE analysis. L: left; R: right; BA: Brodmann area.

Cluster	Volume (mm^3^)	Weighted Center (X,Y,Z)	Maximum ALE Value (X,Y,Z)	ALE Value (10^−2^)	Anatomical Label	BA
1	31,928	−50.8, −9.3, 14.7	−54, −16, 2	2.39	L Superior Temporal Gyrus	22
			−48, −4, 32	1.67	L Precentral Gyrus	6
			−50,−4,42	1.43	L Precentral Gyrus	4
			−32, 20, −2	1.34	L Claustrum	/
			−42, 24, 16	1.26	L Middle Frontal Gyrus	46
			−54, 8, 22	1.46	L Inferior Frontal Gyrus	9
			−58, −34, −4	0.92	L Middle Temporal Gyrus	21
			−48, 20, −12	0.90	L Inferior Frontal Gyrus	47
			−54, −36, 22	0.84	L Insula	13
			−54, −22, 22	0.84	L Postcentral Gyrus	40
			−30, −12, 8	0.71	L Putamen	/
			−66, −44, −12	0.66	L Middle Temporal Gyrus	20
2	10,616	−3.5, −73.2, −14.5	12, −76, −14	1.20	R Cerebellum Declive	/
			−8, −82, −14	1.11	L Cerebellum Declive	/
			−10, −66, −20	1.06	L Cerebellum Fastigium	/
			−22, −64, −26	0.99	L Cerebellum Culmen	/
			−22, −74, −26	0.94	L Cerebellum Uvula	/
			−6, −94, −4	0.91	L Lingual Gyrus	17
			−20, −64, −38	0.66	L Cerebellum Tonsil	/

**Table 3:** PWS group clusters found by ALE analysis.

Cluster	Volume (mm^3^)	Weighted Center (X,Y,Z)	Maximum ALE Value (X,Y,Z)	ALE Value (10^−2^)	Anatomical Label	BA
1	47,408	46.1, −2.5, 10.2	56, −2, 28	2.70	R Postcentral Gyrus	6
			46,22,10	1.95	R Inferior Frontal Gyrus	45
			60, −6, −2	1.92	R Superior Temporal Gyrus	22
			50, 6, 8	1.79	R Precentral Gyrus	44
			20, −2, −2	1.65	R Globus Pallidus	/
			46, −26, 12	1.55	R Transverse Temporal Gyrus	41
			42, −6, 14	1.54	R Insula	13
			18, 12, −14	1.76	R Putamen	/
			66, −2, 20	1.51	R Precentral Gyrus	4
			14, 8, 2	1.38	R Caudate	/
			42, −14, −6	1.38	R Claustrum	/
			50, 16, −10	1.23	R Inferior Frontal Gyrus	47
			44, −18, 44	1.22	R Postcentral Gyrus	3
			54, −4, −14	0.94	R Superior Temporal Gyrus	38
			56, −32, 0	0.94	R Middle Temporal Gyrus	21
			58, −30, 32	0.83	R Inferior Parietal Lobule	40
2	36,528	−46.8, −9.2, 15	−52, −8, 30	2.53	L Precentral Gyrus	6
			−56, −4, 20	1.73	L Precentral Gyrus	4
			−24, 2, 6	1.62	L Putamen	/
			−66, −20, −2	1.56	L Superior Temporal Gyrus	22
			−48, −42, 20	1.43	L Superior Temporal Gyrus	13
			−38, 20, 4	1.43	L Insula	13
			−12, −16, 10	1.41	L Thalamus	/
			−58, −32, 12	1.37	L Superior Temporal Gyrus	42
			−46, 26, 4	1.32	L Inferior Frontal Gyrus	13
			−52, 36, 2	1.23	L Inferior Frontal Gyrus	46
			−56, 8, 24	0.99	L Inferior Frontal Gyrus	9
			−52, −16, 46	0.84	L Postcentral Gyrus	2
			−50, −26, 32	0.83	L Inferior Parietal Lobule	40
			−58, −48, 22	0.77	L Supramarginal Gyrus	40
			−50, 24, 14	0.70	L Inferior Frontal Gyrus	45
3	11,744	−0.2, 6.2, 48.8	−6, 4, 52	2.02	L Medial Frontal Gyrus	6
			0,4, 48	1.76	L Cingulate Gyrus	24
			6, 4, 50	1.59	R Medial Frontal Gyrus	6
			6, −8, 48	1.05	R Paracentral Lobule	31
			−14, 20, 48	0.93	L Superior Frontal Gyrus	6
			0, −14, 48	0.77	R Paracentral Lobule	31
4	11,664	−16.9, −61.7, −17.6	−6, −66, −18	2.00	L Cerebellum Declive	/
			2, −38, −8	1.20	L Cerebellum Culmen	/
			−36, −72, −26	1.18	L Cerebellum Uvula	/
			10, −52, −18	1.16	R Cerebellum Culmen	/
			−44, −72, −12	1.05	L Fusiform Gyrus	19
			8, −48, −10	0.89	R Cerebellum Cerebellar Lingual	/
			−14, −36, 0	0.85	L Parahippocampal Gyrus	27

**Table 4:** Overlapping clusters between PWNS and PWS group found by ALE analysis.

Cluster	Volume (mm^3^)	Weighted Center (X,Y,Z)	Maximum ALE Value (X,Y,Z)	ALE Value (10^−2^)	Anatomical Label	BA
1	6536	−50, −5.9, 28	−14, −36, 0	1.56	L Precentral Gyrus	6
			−56, 8, 22	0.98	L Inferior Frontal Gyrus	9
2	2600	−59.6, −31.2, 12.2	−58, −34, 12	1.57	L Superior Temporal Gyrus	22
			−62, −34, 18	1.27	L Superior Temporal Gyrus	42
3	1152	−8.9, −66.4, −19.2	−10, −66, −20	1.01	L Cerebellum Fastigium	/
4	368	−34.4, 19.5, 4	−34, 20, 2	1.00	L Insula	13
5	248	−26.4, −67.7, −17.3	−26, −68, −16	0.67	L Cerebellum Declive	/

## Data Availability

The data presented in this study are available on request from the corresponding author.
